# Pharmacokinetics and Bioequivalence Evaluation of Cyclobenzaprine Tablets

**DOI:** 10.1155/2013/281392

**Published:** 2013-09-16

**Authors:** Tatiane Maria de Lima Souza Brioschi, Simone Grigoleto Schramm, Eunice Kazue Kano, Eunice Emiko Mori Koono, Ting Hui Ching, Cristina Helena dos Reis Serra, Valentina Porta

**Affiliations:** Faculty of Pharmaceutical Sciences, University of São Paulo, 580 Avenida Prof. Lineu Prestes, 05508900 São Paulo, SP, Brazil

## Abstract

The purpose of this study was to investigate cyclobenzaprine pharmacokinetics and to evaluate bioequivalence between two different tablet formulations containing the drug. An open, randomized, crossover, single-dose, two-period, and two-sequence design was employed. Tablets were administered to 23 healthy subjects after an overnight fasting and blood samples were collected up to 240 hours after drug administration. Plasma cyclobenzaprine was quantified by means of an LC-MS/MS method. Pharmacokinetic parameters related to absorption, distribution, and elimination were calculated. Cyclobenzaprine plasma profiles for the reference and test products were similar, as well as absorption pharmacokinetic parameters AUC (reference: 199.4 ng**∗**h/mL; test: 201.6 ng**∗**h/mL), C_max_ (reference: 7.0 ng/mL; test: 7.2 ng/mL), and *T*_max_ (reference: 4.5 h; test: 4.6 h). Bioequivalence was evaluated by means of 90% confidence intervals for the ratio of AUC (93%–111%) and *C*_max_ (93%–112%) values for test and reference products, which were within the 80%–125% interval proposed by FDA. Cyclobenzaprine pharmacokinetics can be described by a multicompartment open model with an average rapid elimination half-life (*t*_(1/2)*β*_) of 3.1 hours and an average terminal elimination half-life (*t*_(1/2)*γ*_) of 31.9 hours.

## 1. Introduction

Cyclobenzaprine, 3-(5H-dibenzo[a,d]cyclohepten-5-ylidene)-N,N-dimethyl-1-propanamine, is classified as a skeletal muscle relaxant and is one of the most commonly prescribed agents for the management of musculoskeletal pain [1, 2]. It was first synthesized in 1961, and, in 1977, the 10 mg dose was approved as an adjunct to rest and physical therapy for the relief of muscle spasm associated with acute painful musculoskeletal conditions, influencing both gamma (*γ*) and alpha (*α*) motor systems [1–4]. However, the sedation produced at this dose limited its use until 2003, when the efficacy of cyclobenzaprine hydrochloride 5 mg was established in two well-designed clinical studies [2, 5].

Cyclobenzaprine is structurally similar to tricyclic antidepressants (TCAs) and was first studied as an antidepressant with regard to efficacy and safety. The exact mechanism of action is unknown, but it is presumed to work at the brainstem level of the central nervous system rather than the spinal cord level [5, 6]. Its chemical similarity to TCAs explains its anticholinergic activity and main adverse effects [5–7]. Tolerability of cyclobenzaprine hydrochloride 5 mg and 10 mg is similar, but the 5 mg dose is associated with lower incidence of somnolence (29% versus 38%) and dry mouth (21% versus 32%). Other adverse effects commonly seen with both doses include fatigue (both, 6%) and headache (both, 5%). It can also cause electrocardiogram QT interval prolongation and may raise intraocular pressure [2, 6]. As a consequence of its adverse effects, cyclobenzaprine should be avoided in the elderly, in patients with arrhythmias, cardiac conduction disturbances, heart block, heart failure, or recent myocardial infarction, and in patients with glaucoma [6].

The recommended dose of cyclobenzaprine hydrochloride for relief of muscle spasm for most patients is 5 mg t.i.d. (immediate-release tablets), but it may be increased to 10 mg t.i.d. (immediate-release tablets) [2, 6]. Cyclobenzaprine has also been evaluated in fibromyalgia syndrome (FMS), at doses that usually begin at 10 mg near bedtime and may achieve 30 mg, either at night or divided during the day [8].

After 10 mg immediate-release tablet oral administration, cyclobenzaprine is absorbed with estimates of mean bioavailability ranging from 33% to 55% [9–11]. Approximately 93% of the drug is bound to plasma proteins [12]. Over the dose range 2.5 mg to 10 mg, cyclobenzaprine exhibits linear pharmacokinetics [11]. The drug is extensively metabolized by cytochromes P-450 3A4 and 1A2 and is excreted by kidney primarily as glucuronides [12, 13]. Its elimination half-life is increased in the elderly and in patients with hepatic impairment [6]. Cyclobenzaprine pharmacokinetics is not well defined. Some authors describe the highly variable elimination half-life, ranging from 8 to 37 hours [6, 11], while Darwish and coworkers describe the average values of 30–35 hours and lower variability for cyclobenzaprine elimination half-life [14–17].

The bioavailability of a drug product is defined as the rate and extent to which the active ingredient or therapeutic moiety is absorbed and becomes available at the site of drug action. Two drug products are considered to be bioequivalent if they are pharmaceutical equivalents (i.e., similar dosage forms made, perhaps, by different manufacturers) or pharmaceutical alternatives (i.e., different dosage forms) and if their rates and extents of absorption do not show a significant difference when administered at the same molar dose of the therapeutic moiety under similar experimental conditions [18]. Bioequivalence or comparative bioavailability has gained increasing attention during the last 40 years after it became evident that marketed products having same amounts of the same drug may exhibit marked differences between their therapeutic responses. In many instances, these differences were correlated successfully to dissimilar drug blood levels caused mainly by impaired absorption [19].

The purpose of this study was to investigate cyclobenzaprine pharmacokinetics and to evaluate bioequivalence between two different tablet formulations containing the drug.

## 2. Materials and Methods

### 2.1. Samples

Samples of two different cyclobenzaprine 10 mg immediate-release tablets (reference and test products) were used.

### 2.2. Cyclobenzaprine Quantification in Human Plasma

Several methods have been used for cyclobenzaprine quantification in plasma samples. Techniques used are thin-layer chromatography [20], gas-liquid chromatography with nitrogen detector [21], capillary gas chromatography with either flame-ionization or nitrogen-selective detection [22], and high-performance liquid chromatography with tandem mass spectrometric or ultraviolet detection [15, 23, 24]. All of these methods bear disadvantages which hinder their application on pharmacokinetics and bioequivalence studies, related to the use of not commercially available internal standard [15, 23] or to a low sensitivity [20–22, 24].

As part of this study, an LC-MS/MS method was developed and validated for cyclobenzaprine quantification in plasma samples.

#### 2.2.1. Chemicals

Cyclobenzaprine and amitriptyline (internal standard (IS)) were kindly provided by Apsen Farmacêutica S/A (São Paulo, Brazil). Acetonitrile (HPLC grade), methyl tert-butyl ether (HPLC grade), formic acid (ACS grade), and ammonium acetate (ACS grade) were from Merck (Darmstadt, Germany). Purified water was prepared with a Milli-Q Academic System (Millipore Co., USA).

#### 2.2.2. Extraction Procedure

Sample preparation was performed by extracting plasma cyclobenzaprine with an organic solvent. 1000 *μ*L of plasma and 25 *μ*L of amitriptyline (IS) solution (1000 ng/mL in methanol) were added to 10 mL glass tubes. The samples were extracted with 4.0 mL of methyl tert-butyl ether by vortex-mixing for 1 min. After centrifugation for 10 min at 3500 rpm, samples were frozen, and the organic layer was filtered through a Millex GV 0.45 *μ*m filter unit into a 10 mL conical glass tube and was evaporated at 40°C under a nitrogen stream. Residues were reconstituted with 500 *μ*L of mobile phase, vortex-mixed for 30 s, and transferred to auto-sampler vials, and 50 *μ*L was injected into the LC-MS/MS system.

#### 2.2.3. LC-MS/MS System

LC-MS/MS system consisted of Shimadzu LC-10ADvp pump, DGU-14A degasser, SIL-10ADvp autosampler, CTO-10ADvp column oven, SCL-10ADvp system controller (Shimadzu Scientific Instruments, Kyoto, Japan), and Micromass Quattro triple quadrupole mass spectrometer (Waters, Milford, CT, USA).

Mobile phase was a mixture of acetonitrile and 0.01 M ammonium acetate buffer (90 : 10, v/v), with formic acid 0.1%. Elution was performed at a flow-rate of 0.35 mL/min through a Phenomenex Luna C18, 150 × 4.6 mm column at room temperature.

Cyclobenzaprine was monitored and quantified through mass spectrometric detection using multiple reaction monitoring (MRM) of the transitions *m*/*z* 276.6→216.4 and 278→218 for cyclobenzaprine and IS, respectively. Positive ion mode electrospray ionization (ESI) was used. Mass spectrometer source temperature was 100°C. Desolvation gas flow was set at 405 L/hr, and desolvation temperature was set at 350°C. Capillary and cone voltages were 3.0 kV and 35 kV, respectively, and collision energy was 25 eV. Data processing was performed on Mass Lynx 3.5 Software.

#### 2.2.4. Method Validation

Method validation was accomplished through determination of specificity, lower limit of quantification (LLOQ), linearity, recovery, precision, accuracy, and stability of plasma samples and reconstituted residues [25–27].

### 2.3. Pharmacokinetics and Bioequivalence Study

The study protocol was approved by the local ethics committee. Twenty-six healthy volunteers (13 males and 13 females), with average age, weight, and height of 32 years, 65 kg, and 167 cm, respectively, were enrolled. All volunteers gave written informed consent to participate in the study. Volunteers were nonsmokers, had no history of heart, kidneys, neurological, or metabolic diseases, had no history of drug hypersensitivity, and were not undergoing any pharmacological treatment, and female volunteers were not pregnant, as confirmed by physical examination, electrocardiogram, and blood and urine analyses.

The study was an open, single-dose, randomized, two-period, two-sequence, and crossover trial with a 30-day washout between the periods. During the first period, after an overnight fasting, volunteers from group A received a 10 mg cyclobenzaprine tablet of reference product, while volunteers from group B received a 10 mg cyclobenzaprine tablet of test product, with 200 mL of water. Volunteers received standard lunch, afternoon snack, and dinner, respectively, 4, 7, and 10 hours after drug administration. During the second period, the procedure was repeated on the groups in reverse.

Venous blood samples were collected through indwelling catheters to heparinized tubes at 0 (before dose), 1, 2, 3, 4, 5, 6, 7, 8, 10, 12, 24, 48, 72, 96, 144, 192, and 240 hours after drug administration. Samples were immediately centrifuged at 3000 rpm for 15 min, and the plasma was stored at −20°C until cyclobenzaprine quantification.

### 2.4. Pharmacokinetics and Statistical Analysis

Pharmacokinetic parameters related to absorption, distribution, and elimination of the drug were calculated using Microsoft Office Excel 2007 for Windows 7: AUC (area under concentration—time curve), *C*_max⁡_ (peak drug concentration), *T*_max⁡_ (time to reach *C*_max⁡_), *V*_*d*_ (distribution volume), Cl (drug clearance after oral administration), *K*_*β*_ (rapid elimination rate constant), *t*_(1/2)*β*_ (rapid elimination half-life), *K*_*γ*_ (terminal elimination rate constant), *t*_(1/2)*γ*_ (terminal elimination half-life), and MRT (mean residence time).

AUC was calculated using the linear trapezoidal rule. Cyclobenzaprine plasma concentrations below the lower limit of quantification (LOL) were achieved at or prior to 240 hr after drug administration for all volunteers and both formulations, so AUC was calculated considering drug concentration values below LOL as zero and no distinction was made between AUC_0-t_ (area under concentration—time curve from time zero to time *t*, where *t* is the last point with measurable concentration) and AUC_0-*∞*_ (area under concentration-time curve from time zero to infinite). *C*_max⁡_ and *T*_max⁡_ were obtained directly from experimental data without interpolation. *K*_*β*_ and *K*_*γ*_ were determined by the method of residuals. Half-lives *t*_(1/2)*β*_ and *t*_(1/2)*γ*_ were calculated by means of the equation *t*_(1/2)_ = 0,693/*K*. Drug clearance after oral administration (Cl) was determined directly from the plasma drug concentration-time curve by Cl = *DF*/AUC, where *D* is the administered dose and *F* is the fraction of drug absorbed after oral administration considered as 0,55 [9–11]. Volume of distribution (*V*_*d*_) was obtained by the equation *V*_*d*_ = Cl/*K*_*β*_. MRT was calculated by the equation MRT = AUMC/AUC, where AUMC is the area under the first moment-time curve [28]. 

Statistical and bioequivalence analysis was performed using SPSS version 15.0 for Windows and SAS version 6.12. Bioequivalence between the products was determined by calculating 90% confidence intervals (90% CI) for the ratio of *C*_max⁡_ and AUC values for test and reference products, using logarithmic transformed data. Analysis of variance (ANOVA) was used to assess product, group, and period effects. The products were considered bioequivalent if the 90% CI for *C*_max⁡_ and AUC fell within the 80%–125% interval proposed by most regulatory agencies like the Food and Drug Administration (FDA, USA) and the European Medicines Agency (EMA).

Parametric (paired *t*-test) and nonparametric (Wilcoxon) statistic tests were employed to compare distribution- and elimination-related pharmacokinetic parameters values for test and reference products. Absence of significant differences would allow the calculation of average values for these parameters based on pooled data from both products [29].

## 3. Results and Discussion

### 3.1. Cyclobenzaprine Quantification in Human Plasma

The LC-MS/MS method used for cyclobenzaprine quantification provided the sensitivity, specificity, and high sample throughput required for pharmacokinetic and bioequivalence studies. 

**Figure 1:**
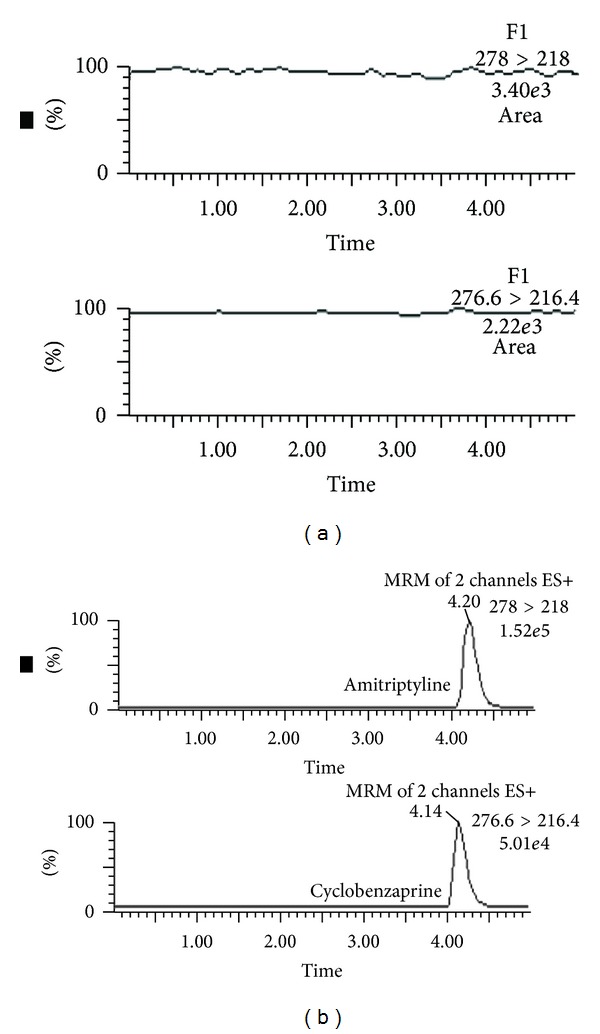
Chromatograms of (a) blank human plasma and (b) plasma from healthy volunteer following oral administration of cyclobenzaprine 10 mg, spiked with amitriptyline (IS).

Retention times of cyclobenzaprine and IS (amitriptyline) were 4.1 and 4.2 minutes, respectively, and no interfering peaks from endogenous components of blank plasma samples were observed (Figure 1).

The method exhibited a reliable linear response in the concentration range from 0.25 to 15 ng/mL (*y* = 0.0648*x* − 0.0014; *r* ^2^ = 0.9979).

LLOQ was 0.25 ng/mL in plasma, with acceptable accuracy and precision of 104.4% and 9.2%, respectively. 

Mean extraction recovery values were 92.8% for cyclobenzaprine and 94.4% for IS in plasma samples.

Intra-assays accuracy and precision ranged from 90.6% to 96.7% and from 3.1% to 3.7%, respectively, while interassays accuracy and precision ranged from 95.2% to 98.8% and from 5.5% to 6.9%, respectively.

Plasma samples were stable during three freezing-thaw cycles as well during storage at −20°C for 120 days. Plasma samples residues from the extraction procedure, reconstituted with mobile phase for chromatographic analysis, were stable at room temperature for 48 hours.

### 3.2. Pharmacokinetics and Bioequivalence Study

**Figure 2:**
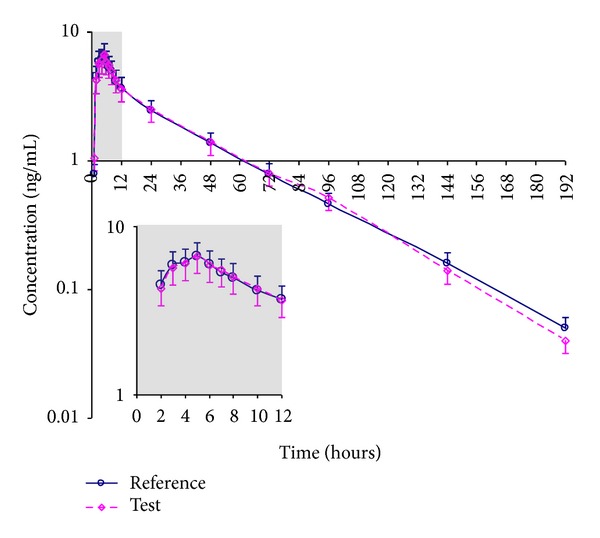
Average plasma concentrations of cyclobenzaprine after oral administration of single oral doses (10 mg) of reference and test products to 23 healthy volunteers. Bars indicate mean standard errors (upper bars for reference product and lower bars for test product).

Twenty-three volunteers (12 males and 11 females) completed the study.  Figure 2 shows average concentration versus time curves after administration of reference and test products to the volunteers. Mean pharmacokinetic parameters related to drug absorption derived from these curves are presented in Table 1. The average plasma decay curves obtained for the test and reference products were similar as were pharmacokinetic parameters.

Previous studies reported *C*_max⁡_ values of 26.7 ng/mL and 29.6 ng/mL following oral administration of 40 mg of cyclobenzaprine (four 10 mg cyclobenzaprine tablets) to healthy volunteers [10, 20]. These values are comparable with those obtained in the present study, 7.0 ng/mL for reference product and 7.2 ng/mL for test product after oral administration of 10 mg of cyclobenzaprine. Time to reach the maximum concentration (*T*_max⁡_) in this study (4.5 hr for reference product and 4.6 hr for test product) was also similar to that obtained in previous studies, which described *T*_max⁡_ values ranging from 3.8 to 6.0 hr [11, 20].

Multivariate analysis accomplished through analysis of variance (ANOVA) for assessment of product, group, and period effects revealed the absence of any of these effects.

90% confidence intervals for the ratio of *C*_max⁡_ (93%–112%) and AUC (93%–111%) values for the test and reference products are within the 80%–125% interval, thus fulfilling the bioequivalence criteria adopted by FDA (USA), EMA (European Union), and ANVISA (Brazil).

Comparison of the values of distribution and elimination pharmacokinetic parameters after reference and test products administration revealed no significant differences between them. As a consequence, average values for *K*_*β*_, *K*_*γ*_, *t*_(1/2)*β*_, *t*_(1/2)*γ*_, Cl, *V*_*d*_, and MRT were obtained using pooled data from both products to describe cyclobenzaprine pharmacokinetics (Table 2).

The large variability observed in cyclobenzaprine pharmacokinetic parameters is probably a consequence of heterogeneous uptake in the gastrointestinal tract, due to low drug permeability [30]. Drug permeability and drug solubility are the two main key biopharmaceutical parameters which control drug absorption. A biopharmaceutical classification system (BCS) based on these parameters was proposed by Amidon and coworkers [31]. According to the BCS, drugs can be divided into high/low solubility-permeability classes, with different expectations regarding their oral absorption. The solubility classification of a drug in the BCS is based on the highest dose strength in an immediate-release drug product. A drug substance is considered highly soluble when the highest dose strength is soluble in 250 mL or less of aqueous media over the pH range of 1–7.5; otherwise, the drug substance is considered poorly soluble. The volume estimate of 250 mL is derived from typical bioequivalence study protocols that prescribe administration of a drug product to fasting human volunteers with a glass of water [32]. The permeability classification is based directly on the extent of intestinal absorption of a drug substance in humans or indirectly on the measurements of the rate of mass transfer across the human intestinal membrane. A drug substance is considered highly permeable when the extent of intestinal absorption is determined to be 90% or higher. Otherwise, the drug substance is considered to be poorly permeable [32]. Cyclobenzaprine is poorly permeable, since its intestinal absorption after oral administration is estimated to be between 33% and 55% [9–11]. Solubility of cyclobenzaprine in water at 37°C is 664 mg/mL [33], but solubility data in other media over the pH range of 1–7.5 are not available. Therefore, cyclobenzaprine could be a class III (high solubility and low permeability) or a class IV (low solubility and low permeability) drug. Both the rate and extent of drug absorption may be highly variable for class III drugs due to variable gastrointestinal transit, luminal contents, and membrane permeability rather than dosage form factors [31]. Class IV drugs present significant problems for effective oral delivery [31]. Cyclobenzaprine extensive distribution to tissue and binding to acid glycoprotein, which plasma concentration increases as part of acute-phase reaction to illness [11], may also contribute to the large pharmacokinetics variability.

Drug concentration-time curve for cyclobenzaprine after oral administration to healthy volunteers declines biexponentially, as can be observed in Figure 2. This could indicate that cyclobenzaprine follows the pharmacokinetics of a two-compartment model, with a distribution phase (first exponential decay) and an elimination phase (second exponential decay). However, the distribution process for drugs that follow bicompartmental models is usually rapid, and this is not the case for the first exponential decay of cyclobenzaprine, which is prolonged for at least seven hours, taking place between 5 hours (*T*_max⁡_) and 12 hours after drug administration. Considering this, there is a higher possibility that the first and second exponential decays relate to beta- and gamma-elimination phases, respectively, and thus cyclobenzaprine pharmacokinetics could be described as a three-compartment model. Beta-elimination phase corresponds to rapid elimination from the central compartment with an elimination half-life of 3.1 h, while gamma-elimination phase corresponds to slower elimination from a deep compartment with a terminal elimination half-life of 31.9 h. Non-observation of distribution phase can be explained by two reasons: drug distribution is faster than drug absorption and cyclobenzaprine becomes distributed during absorption, or the sampling intervals after *T*_max⁡_ were too large to detect the distribution process. 

## 4. Conclusion

Pharmacokinetics and statistical results indicate that the two formulations of cyclobenzaprine are bioequivalent in their rate and extent of absorption and that cyclobenzaprine pharmacokinetics can be described by a multicompartment open model with an average rapid elimination half-life (*t*_(1/2)*β*_) of 3.1 hours and an average terminal elimination half-life (*t*_(1/2)*γ*_) of 31.9 hours.

## Figures and Tables

**Table 1:** Pharmacokinetic parameters related to absorption and 90% confidence intervals for *C*_max⁡_ and AUC after single-dose oral administration of one tablet (10 mg of cyclobenzaprine) of reference and test products to 23 healthy volunteers.

	Test (average ± SD)	Reference (average ± SD)	90% CI
*C*_max⁡_ (ng/mL)	7.2 ± 2.5	7.0 ± 2.2	93–112%
AUC (ng∗h/mL)	201.6 ± 95.5	199.4 ± 93.7	93–111%
*T*_max⁡_ (h)	4.6 ± 1.3	4.5 ± 1.3	

AUC: area under concentration—time curve; *C*_max⁡_: peak drug concentration; *T*_max⁡_: time to reach *C*_max⁡_; SD: standard deviation; 90% CI: 90% confidence interval.

**Table 2:** Pharmacokinetic parameters after oral administration of 10 mg cyclobenzaprine tablets to healthy volunteers.

	AUC (ng∗h/mL)	*C*_max⁡_ (ng/mL)	*T*_max⁡_ (h)	*V*_*d*_ (L)	Cl (L/h)	K_β_ (h^−1^)	t_(1/2)β_ (h)	K_γ_ (h^−1^)	t_(1/2)γ_ (h)	MRT (h)
Average	200.5	7.1	4.5	146.2	33.1	0.247	3.1	0.024	31.9	34.7
SD	93.6	2.3	1.3	81.6	14.0	0.088	1.0	0.008	8.9	10.4
CV (%)	46.7	33.0	29.2	55.8	42.2	35.8	31.4	33.2	28.0	30.0
Median	182.8	6.8	5.0	118.2	30.1	0.232	3.0	0.022	31.1	33.3
Minimum	84.3	3.2	2.0	34.2	10.7	0.137	1.3	0.013	14.2	16.3
Maximum	512.9	11.4	7.0	354.5	65.3	0.524	5.1	0.049	51.7	56.3

AUC: area under concentration—time curve; *C*_max⁡_: peak drug concentration; *T*_max⁡_: time to reach *C*_max⁡_; *V*_*d*_: distribution volume; Cl: drug clearance after oral administration; *K*_*β*_: rapid elimination rate constant; *t*_(1/2)*β*_: rapid elimination half-life; *K*_*γ*_: terminal elimination rate constant; *t*_(1/2)*γ*_: terminal elimination half-life; MRT: mean residence time; SD: standard deviation; CV: coefficient of variation.
